# Mitochondrial genome sequence and gene order of *Sipunculus nudus *give additional support for an inclusion of Sipuncula into Annelida

**DOI:** 10.1186/1471-2164-10-27

**Published:** 2009-01-16

**Authors:** Adina Mwinyi, Achim Meyer, Christoph Bleidorn, Bernhard Lieb, Thomas Bartolomaeus, Lars Podsiadlowski

**Affiliations:** 1 Institut für Zoologie, Freie Universität Berlin, Koenigin-Luise-Str. 1-3, 14195 Berlin, Germany; 2 Institut für Zoologie, Johannes Gutenberg Universität Mainz, Müllerweg 6, 55099 Mainz, Germany; 3 Institut für Biochemie und Biologie, Universität Potsdam, Karl-Liebknecht-Str. 24-25, 14476 Golm, Germany; 4 Institut für Evoilutionsbiologie & Ökologie, Universität Bonn, An der Immenburg 1, 53121 Bonn, Germany

## Abstract

**Background:**

Mitochondrial genomes are a valuable source of data for analysing phylogenetic relationships. Besides sequence information, mitochondrial gene order may add phylogenetically useful information, too. Sipuncula are unsegmented marine worms, traditionally placed in their own phylum. Recent molecular and morphological findings suggest a close affinity to the segmented Annelida.

**Results:**

The first complete mitochondrial genome of a member of Sipuncula, *Sipunculus nudus*, is presented. All 37 genes characteristic for metazoan mtDNA were detected and are encoded on the same strand. The mitochondrial gene order (protein-coding and ribosomal RNA genes) resembles that of annelids, but shows several derivations so far found only in Sipuncula. Sequence based phylogenetic analysis of mitochondrial protein-coding genes results in significant bootstrap support for Annelida *sensu lato*, combining Annelida together with Sipuncula, Echiura, Pogonophora and Myzostomida.

**Conclusion:**

The mitochondrial sequence data support a close relationship of Annelida and Sipuncula. Also the most parsimonious explanation of changes in gene order favours a derivation from the annelid gene order. These results complement findings from recent phylogenetic analyses of nuclear encoded genes as well as a report of a segmental neural patterning in Sipuncula.

## Background

Molecular sequence analysis has become the method of choice to address phylogenetic questions. The applied techniques improve continually and the rapidly growing amount of available data helps to broaden our knowledge of phylogenetic relationships within the animal kingdom. Nevertheless, different molecular datasets often show conflicting phylogenetic signals, so that results relying on just one dataset may be interpreted with caution [1]. Unlike nuclear DNA, the mt-genome of animals is normally rather small and simply structured: haploid, without or only few non-coding segments, repetitive regions and transposable elements. Derived from endosymbiotic bacteria only a few genes are retained in the mitochondrial genomes of Bilateria: 13 protein subunits (*nad1-6, nad4L, cox1-3, cob, atp6/8*), 2 ribosomal RNAs (*rrnL, rrnS*) and 22 tRNAs are found encoded on a circular doublestranded DNA molecule sized about 15 kb [2,3]. As such sequencing and annotation of mt-genomes is much easier and faster than analysing nuclear genomes, making mt-genomes one of the commonly used sources of sequence data for phylogenetic analyses. Apart from sequence data other features of the genome may contain phylogenetic information, too. Taxon-specific gene order often remains identical over long periods of time [4-6]. Simultaneously, the intra-taxonomic variances of these characteristic orders are quite distinctive and convergent changes in the positioning of single genes are rather unlikely, due to the vast number of possible combinations [7]. Thus changes in the mitochondrial gene order have proved to be valuable tools in phylogenetic analyses [8-10]. Less often secondary structures of tRNAs or rRNAs show distinct differences between taxa (e.g. loss of a stem/loop region) and hence may also contribute to a phylogenetic analysis [11].

The taxon Sipuncula (peanut worms) comprises about 150 species, being found in all water depths of different marine habitats. The hemisessile organisms dwell in mud and sand, but settle also in empty mollusc shells or coral reef clefts for instance. Their body shows no segmentation, but a subdivision into a posterior trunk and an anterior introvert that can be fully retracted into the trunk is observeable [12]. Fossils that date back into the later cambrian [13] suggest that sipunculans have undergone little morphologically change over the past 520 Myr. The monophyly of this morphologically uniform taxon is well founded by morphological [14] and molecular data [15]. However, the phylogenetic position within Bilateria was highly disputed. Based on morphological characters, very different phylogenetic positions of Sipuncula were discussed. Early in history an affinity to Echinodermata, especially holothurians was mentioned and later again propagated by Nichols [16], but with little acceptance from other authors. Scheltema [17] proposed a close relationship to molluscs based on the presence of the so calles "molluscan cross" organization of micromeres during spiral cleavage. The usefulness of this character for phylogenetic inference was neglected by Malaskova [18]. Other analyses found Sipuncula to be sister group of Mollusca, Annelida and Arthropoda [19], Articulata (Annelida and Arthropoda) [14], Echiura [20], Mollusca [21], Annelida [22] or Annelida+Echiura [23]. More details about the different hypotheses of sipunculid relationships are reviewed in [24].

In contrast to all these studies, molecular analyses of large datasets from 18S/28S data [25], ESTs [26,27] or mitochondrial genome data [28,29] favour an inclusion of Sipuncula into annelids. An implication of this hypothesis is that we have to assume that segmentation has been reduced within Sipuncula [30]. A derivation from segmented ancestors of Sipuncula was recently also supported by a segmental mode of neural patterning in ontogeny [31].

Relationhips within Sipuncula are well investigated [15,24,32-34]. An analysis using combined molecular and morphological data recovered five major clades and supports that *Sipunculus *is the sister group to all other sipunculids [15].

Up to now mt-genome data from Sipuncula was restricted to a partial mtDNA sequence from *Phascolosoma gouldii *[29], comprising only about half of the complete genome. Here we describe the first complete mitochondrial genome for another representative of the Sipuncula, *Sipunculus nudus*. We analyse sequence data in comparison with mitochondrial genomes of various Bilateria to evaluate the phylogenetic position of Sipuncula. In addition we compare gene order among Lophotrochozoa and evaluate the most parsimonious explanation for gene order changes.

## Results and discussion

### Genome organisation

**Figure 1:**
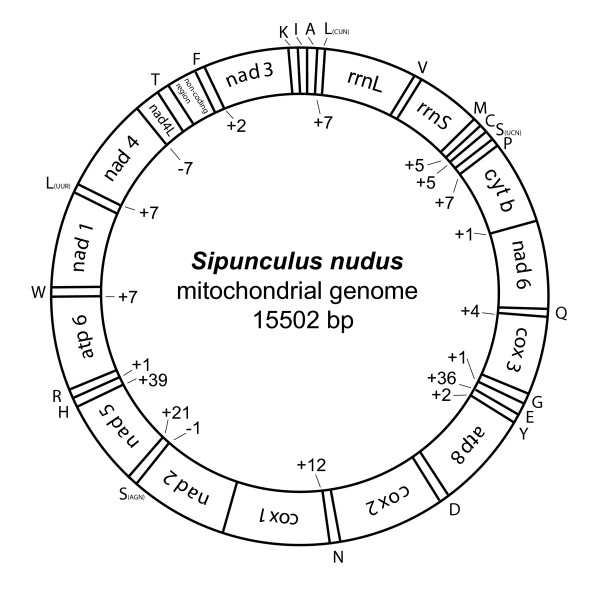
**Circular map of the mitochondrial genome of *Sipunculus nudus***.

The complete mt-genome of *S. nudus *is a circular DNA doublestrand of 15502 bp length. As usual in bilateria, 13 genes coding for different protein subunits and two encoding ribosomal RNA genes were identified. In addition 22 tRNA genes were detected and thus all 37 genes typically present in bilaterian mt genomes, were found (Fig. 1, Table 1). All of these genes are located on the (+)-strand, as is the case in annelid and echiurid mt-genomes. There are two small gene overlaps: one between *nad4L *and *nad4 *(7 bp), the other one between *trnS *(AGN) and *nad2 *(1 bp). The putative control region is 441 bp in length and flanked by *trnF *and *trnT*. Besides the control region 15 other non-coding regions are dispersed over the whole genome, ranging from one to 39 base pairs. The three largest of these are located between *trnY *and *trnE *(35 bp), *trnH *and *nad5 *(39 bp) and *nad5 *and *trnS *(AGN) (21 bp).

**Table 1:** Genome organisation of *Sipunculus nudus*. Complete circular mtDNA has a lenght of 15502 bp.

***Gene***	***Strand***	***Position*** ***(start – end)***	***Length*** ***(nuc.)***	***GC-/AT-*** ***skew***	***Start-*** ***codon***	***Stop-*** ***codon***	***Intergenic bp***
*cox1*	+	1 – 1543	1543	-0.24/-0.07	ATG	TAA	12
*trnN*	+	1556 – 1624	69				0
*cox2*	+	1625 – 2319	695	-0.26/-0.07	ATG	TA	0
*trnD*	+	2320 – 2385	66				0
*atp8*	+	2386 – 2544	159	-0.38/0.12	ATG	TAG	2
*trnY*	+	2547 – 2609	63				36
*trnE*	+	2646 – 2712	67				1
*trnG*	+	2714 – 2780	67				0
*cox3*	+	2781 – 3560	780	-0.27/-0.07	ATG	TAA	4
*trnQ*	+	3565 – 3632	68				0
*nad6*	+	3633 – 4106	474	-0.34/-0.18	ATG	TAG	1
*cob*	+	4108 – 5247	1140	-0.30/-0.06	ATG	TAA	7
*trnP*	+	5255 – 5322	68				0
*trnS*-UCN	+	5323 – 5389	67				5
*trnC*	+	5395 – 5455	61				5
*trnM*	+	5461 – 5527	67				0
*rrnS (12S)*	+	5528 – 6373	846	-0.23/0.18			0
*trnV*	+	6374 – 6442	69				0
*rrnL (16S)*	+	6443 – 7929	1487	-0.26/0.08			0
*trnL*-CUN	+	7930 – 7995	66				7
*trnA*	+	8003 – 8070	68				0
*trnI*	+	8071 – 8139	69				0
*trnK*	+	8140 – 8207	68				0
*nad3*	+	8208 – 8565	358	-0.35/-0.05	ATG	T	2
*trnF*	+	8568 – 8631	64				0
Major NCR	+	8632 – 9072	441	-0.14/0.09			0
*trnT*	+	9073 – 9142	70				0
*nad4L*	+	9143 – 9424	282	-0.43/-0.06	ATG	TAA	-7
*nad4*	+	9418 – 10774	1357	-0.37/-0.02	ATG	T	7
*trnL-*UUR	+	10782 – 10846	65				0
*nad1*	+	10847 – 11789	943	-0.32/-0.09	ATG	T	0
*trnW*	+	11790 – 11855	66				7
*atp6*	+	11863 – 12550	688	-0.41/-0.14	ATG	T	0
*trnR*	+	12551 – 12619	69				1
*trnH*	+	12689 – 12688	68				39
*nad5*	+	12728 – 12727	1698	-0.37/0.01	ATA	TAA	21
*trnS*-AGN	+	14447 – 14518	72				-1
*nad2*	+	14518 – 15502	985	-0.45/-0.13	ATG	T	0

* start and stop position of ribosomal RNA and NCR according to adjacent gene boundaries

The GC-skew [(G-C)/(G+C)] reflects the relative number of cytosin to guanine and is often used to describe the strand-specific bias of the nucleotide composition [35]. In *S.nudus *the complete (+)-strand genome sequence has a clear bias toward Cytosine (GC-skew -0.296). As all genes are coded on (+)-strand, all single gene sequences exhibit a negative GC-skew, too (Table 1), ranging from -0.23 (*rrnS*) to -0.45 (*nad2*). A negative GC-skew is also found in most of the mitochondrial genomes known from annelids, pogonophorans, and myzostomids, with the exception of the annelid *Eclysippe vanelli *[36]. AT-skew of the complete (+)-strand is close to evenness (-0.013) and single gene AT-skews are distributed around evenness with a range between 0.18 (*rrnS*) and -0.18 (*nad6*), see also Table 1. AT content of the complete genome is 54.2%, AT contents of protein-coding and rRNA genes are not much derived from this value, between a minimum of 50,3% (*nad3*) and a maximum of 59,8% (*atp8*).

### Protein coding genes

All but one of the protein subunits begin with start codon ATG, only *nad5 *starts with ATA. Both are prevalent in mitochondrial genomes. The commonly found stop codons TAA and TAG are present, as well as the abbreviated forms TA (*cox2*) and T (*nad1*-*4*, *atp6*). Putative shortened stop codons were already found in other species and are thought to be complemented via post-transcriptional polyadenylation [37].

### Ribosomal RNA genes and control region

The sizes of the ribosomal RNAs (*rrnS*: 846 bp; *rrnL*: 1487 bp) are within the range of their sizes in other animals including molluscs and annelids. The two genes only separated by *trnV*, a feature often found in animals from vertebrates to arthropods, so therefore this represent an ancestral condition. Among annelids and their kin only echiurans (*Urechis caupo*) and myzostomids (*Myzostoma seymourcollegiorum*) differ from that condition in that there is no tRNA gene separating the two ribosomal genes. AT content of ribosomal genes is 50.8% (*rrnS*) and 53.1% (*rrnL*), so well within the range of AT content of protein-coding genes.

### Noncoding regions, putative control region

The putative control region is found between *nad3*/*trnF *on one side and *trnT/nad4L/nad4 *on the other side. While gene order (or protein-coding and rRNA genes) in Annelida is more or less conserved there is a great variation in the position of the control region: (a) Species from Clitellata, Maldanidae and Terebellidae have a major non-coding region between *atp6/trnR *and *trnH/nad5*; (b) in *Orbinia *it is located between *nad4/trnC/trnL2 *and *trnL1/trnM/rrnS*; (c) in *Platynereis *it is found between *cox2/trnG *and *trnV/atp8 *[8,28,36,38]. Such great variability is not found in other taxa like Arthropoda or Vertebrata, where also the control region is found in the same position in different species, when gene order of the rest of the mt-genome is conserved.

In *Sipunculus nudus *the major non-coding region has a size of 441 bp and is clearly more AT rich (66.1%) than the rest of the genome (53.9%). Structural elements know from arthropod mitochondrial control regions [39] are present also in *S. nudus*: (1) a poly-TA(A) stretch of 50 bp including a tenfold TA repeat; (2) a poly-T stretch flanked by purin bases; (3) a GA-rich block of 16 bases length. Although we examined the complete non-coding region intensively by software and by eye, no large stem-loop structure was identified. Such a structure is normally found between the poly-T stretch and the GA rich region in arthropods.

### Transfer RNAs

**Figure 2:**
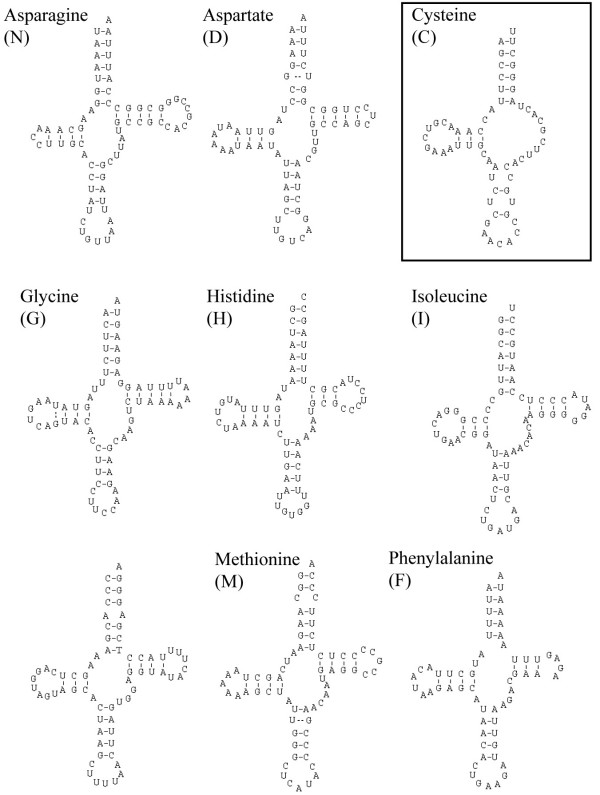
**Secondary structure of tRNAs identified in the mitochondrial genome of *S. nudus***. The best found putative secondary structure of tRNA-Cys (box) seems to be strongly derived, probably non-functional or subject to gene editing. This figure shows only part of the full image, please see also Additional file 1.

All typical 22 tRNAs were detected in the mitochondrial genome of *S. nudus*, their putative secondary structures are depicted in Fig. 2 and Additional file 1. All but three tRNA genes are capable to be folded in the usual cloverleaf structure, consisting of TψC stem and loop, anticodon stem and loop, DHU stem and loop, and the acceptor stem – tRNA-Ser(AGN) and tRNA-Ser(UCN) have no DHU stem. While tRNA-Ser(AGN) shows this feature in many bilaterian mt-genomes, the other one must have changed its secondary structure in the lineage leading to Sipuncula and after the split of its sister group. The putative secondary structure of tRNA-Cys shows no TψC, in addition there are two mismatches in the anticodon stem and an unusual anticodon (ACA), weakening this secondary structure hypothesis. But intensive search for an alternative sequence of tRNA-Cys was not successful, so we stuck with this hypothesis although we cannot rule out that this is a non-functional sequence or subject to gene editing. In several other tRNAs there are mismatches in the acceptor or anticodon stem.

### Mitochondrial gene order

**Figure 3:**
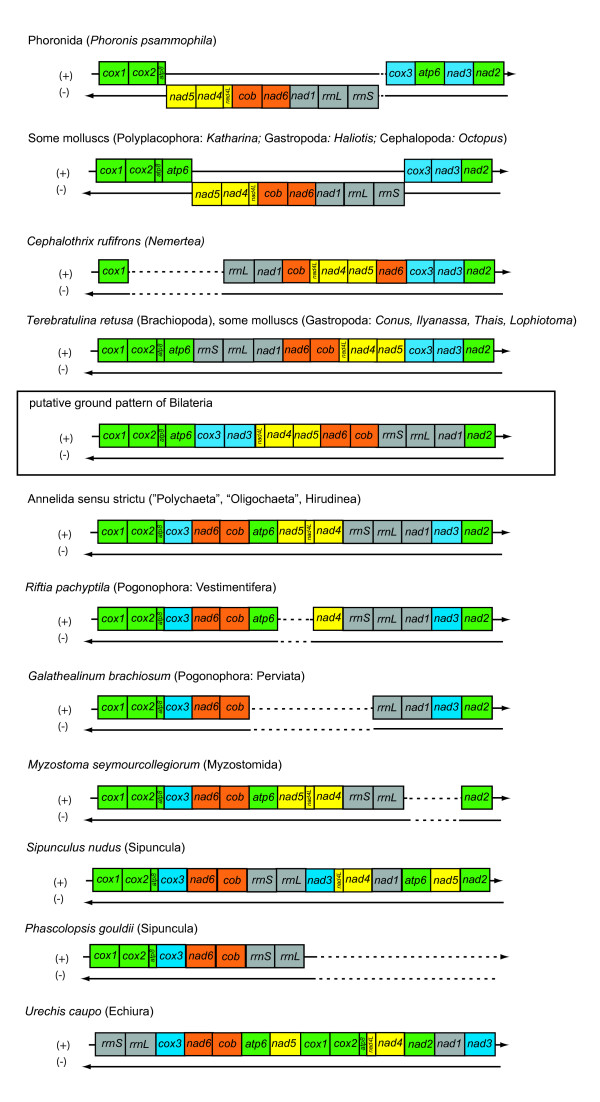
**Comparison of mitochondrial gene order (protein-coding genes and ribosomal RNAs only) of several lophotrochozoan taxa compared and the putative bilaterian ground pattern (according to **[40]**)**. Genome segments from the bilaterian ground pattern are colour coded for a better visualization of differences between gene orders. For complete species names and accession numbers see Table 3.

**Figure 4:**
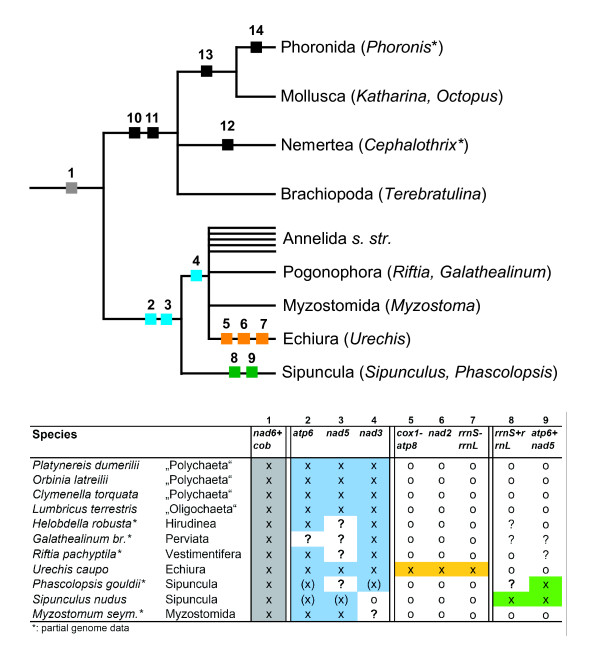
**Cladogram for changes in gene order of lophotrochozoan taxa (only changes in protein-coding and rRNA genes were analysed)**. The translocation of a gene or a gene block is treated as an apomorphic feature (small box) with numbers according to translocated genes in the table below. "x" indicates derived gene positions, circles stand for an unvaried order. "(x)" symbolizes that although the position of the gene is now different there is evidence that it. Questionmarks indicate missing sequence data or putative secondary events complicating the interpretation. Changes not mentioned in the table: (10) translocation of *cox3*/*nad3*; (11) translocation of *rrnS*/*rrnL*/*nad1*; (12) translocation of *nad6*; (13) large inversion of a segment spanning from *rrnS *to *nad5*; (14) translocation of *atp6*. See text for further details.

Fig. 3 shows a comparison of lophotrochozoan mitochondrial gene orders and the ground pattern of Bilateria (as mentioned in [40]). We restrict the discussion of gene order to the protein-coding and rRNA genes, as tRNA genes change their relative position much faster than the former, as seen in gene order comparisons of e.g. annelids [8] or crustaceans [41]. The annelids, pogonophorans and myzostomids do not differ from each other in the relative positions of protein-coding and rRNA genes. Compared to the ground pattern of Bilateria several genes have a different relative position: (1) *nad6/cob *are found right after *cox3*, (2) *atp6 *is found between *cob *and *nad5*, (3) *nad5 *and *nad4L*/*nad4 *have interchanged positions, and (4) *nad3 *is found between *nad1 *and *nad2 *(numbers refer also to hypothesized events in Fig. 4). Mollusca (*Conus textile *[42], *Ilyanassa obsoleta *[43]) and Brachiopoda (*Terebratulina retusa *[44]) show a different pattern, with derived positions for three gene blocks: *rrnS/rrnL/nad1*, *cox3/nad3 *and *nad6*/*cob*. The translocation of *nad6*/*cob *may be explained as a commonly derived feature of Lophotrochozoa, or a subtaxon of it including Mollusca, Phoronida, Brachiopoda, Nemertea, Annelida *s. l*. (including Pogonophora, Echiura and Myzostomida) and Sipuncula (compare Fig. 4). The other translocation events found in annelids and their kin (2.–4.) seem to be restricted to that group. The gene order so far known from Nemertea (*Cephalothrix rufifrons*, partial genome [45]) can be easily derived with one change (translocation of *nad6*) from the pattern of the brachiopod *Terebratulina *and the gene order of Phoronida (*Phoronis psammophila*, partial genome [46]) from that of the mollusc *Katharina tunicata *with only one event (translocation of *atp6*). Much more variation is seen within Mollusca [6,47] and Brachiopoda [48-50] (not shown).

Compared to the Annelida and their kin, the mitochondrial gene order of *Sipunculus nudus *differs clearly: (a) *atp6 *and *nad5 *are found between *nad1 *and *nad2*. This may be interpreted as two events restricted to the sipunculid lineage and independently achieved from the bilaterian or lophotrochozoan ground pattern. But another explanation would be a singular event translocating the block *atp6/nad5 *compared to the annelid ground pattern (No. 8 in Fig. 4); (b) *rrnS/rrnL *found a different position, between *cob *and *nad3 *– this is as well different from the situation in Brachiopoda and Mollusca, so probably another event in the lineage leading to Sipuncula (No. 9 in Fig. 4); (c) *nad3 *is found right after *rrnL *and adjacent to *nad4L*/*nad4*. This is different from its position in annelids, pogonophorans, myzostomids and echiuran taxa and is more similar to the bilaterian ground pattern. Visualized in Fig. 4 the most parsimonious explanation of sipunculid gene order is that Sipuncula share two events with annelids, but lack the translocation of *nad3*. In addition two events have to be assumed in the lineage of Sipunula (*rrnS/rrnL *and *atp6/nad5*, corresponding to 8 and 9 in Fig. 4). Derivation of the *Sipunculus *gene order directly from the bilaterian ground pattern would demand four translocation events (*nad6*/*cob*, *rrnS*/*rrnL*, *atp6*, *nad5*) from which only one is shared with other lophotrochozoan taxa (*nad6/cob*). So this hypothesis is in demand of three additional events instead of two for the "annelid" hypothesis. Derivation of the sipunculid gene order from the brachiopod/mollusc pattern is in demand of five additional events. Therefore the most parsimonious explanation of gene order changes would be that Sipuncula is sister group to a group comprising Annelida *s.str*., Myzostomida, Echiura and Pogonophora.

At first sight gene order of the echiurid *Urechis caupo *[51] is completely different from that of annelids and *Sipunculus*, but the position of *atp6 *between *cob *and *nad5 *and that of *nad3 *adjacent to *nad1 *clearly hint to the derived features postulated for the annelid ground pattern (see b and c in the discussion of annelid gene order above). As well adjacency of *nad6 *to *cox3 *is found in all annelids and *Sipunuculus*. So the gene order of *Urechis *may be derived from the annelid ground pattern, with additional translocations of three genome segments: (a) *cox1/cox2/atp8*, (b) *rrnS/rrnL *and (c) *nad2*.

### Phylogenetic analysis of mitochondrial sequences

**Figure 5:**
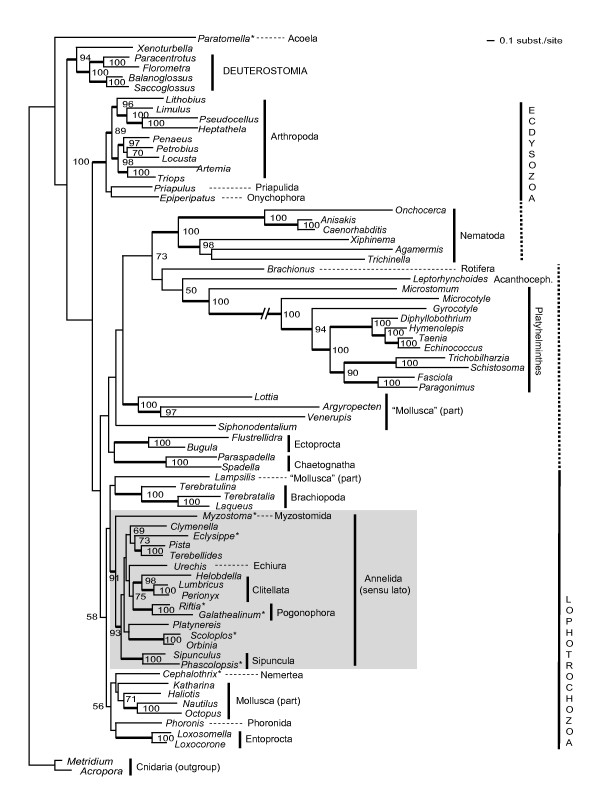
**Best tree from the Maximum Likelihood analysis, inferred from the mitochondrial amino acid data set of 11 protein coding genes (RaxML 7.00, mtREV, G+I, single gene partitions)**. Numbers beneath nodes are ML bootstrap percentages, bold branches indicate bootstrap percentages >85%. See Table 3 for complete species names and accession numbers. Asterisks indicate taxa with incomplete mt-genome information.

The phylogenetic analysis was performed with a concatenated amino acid alignment of 11 protein-coding genes (exept *atp8 *and *nad4L*) from 74 species. Fig. 5 shows the best tree of the Maximum Likelihood analysis with RaxML (mtREV+G+I). A close relationship of *Sipunculus *and *Phascolopsis *and thus monophyletic Sipuncula is well supported (ML bt: 100%). Sipuncula appears to be close related to the classic "Annelida", Echiura and Pogonophora – this assemblage has a bootstrap support of 93%. This assemblage is also other recovered in recent molecular analyses of 18S/28S rRNA and EF1α [25] or EST data [26]. The internal relationships of these taxa are not well resolved by our analysis. With high bootstrap support Clitellata (98%) and Pogonophora (100%) appear monophyletic, while their sister group relationship found only weak support (bootstrap: 75%). Sister group to the Sipuncula/Annelida/Echiura/Pogonophora taxon is Myzostomida (ML bt: 91%), this relationship is also supported by morphological characters and mitochondrial gene order as recently detailed elsewhere [8]. The position of this "Annelida sensu lato" among other Lophotrochozan subtaxa is not well resolved in our analysis.

Probably due to long branch effects, Ecdysozoa and Lophotrochoza appear not to be monophyletic in our analysis. While the former miss Nematoda, the latter miss Platyhelminthes, Ectoprocta, Rotifera, Acanthocephala and some molluscs. All these taxa are associated with long branches and form a probably artificial clade, which was never recovered in analyses with molecular data from nuclear genes or morphological data. Apart from this the most "problematic" taxon are Mollusca, with some taxa (*Lottia*, *Argopecten*, *Venerupis*, *Siphonodentalium*) found clustering with the above mentioned nematode-platyhelminth assemblage, others (*Katharina*, *Haliotis*, *Nautilus*, *Octopus*) clustering with Nemertea, Phoronida and Entoprocta, while *Lampsilis *appears as sister taxon to Brachiopoda.

**Figure 6:**
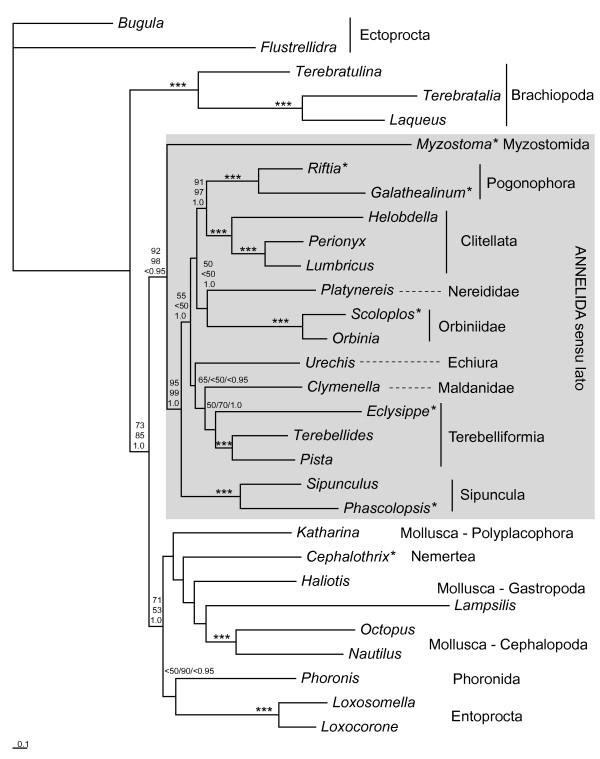
**Best tree from the Maximum Likelihood analysis (RAxML 7.00, mtREV, G+I, single gene partitions) of the reduced taxon set (30 lophotrochozoan species)**. Numbers beneath nodes indicate support (from left to right or up to down, respectively): (1) through RaxML bootstrapping (1000 pseudoreplicates) (2) ML analysis with Treefinder (1000 pseudoreplicates), model mtART+G+I, (3) Bayesian posterior probabilities (model mtREV+G+I). Triple asterisks indicate maximum support from all three analyses (100/100/1.0). See Table 3 for complete species names and accession numbers. Single asterisks indicate taxa with incomplete mt-genome information. Scalebar depicts substitutions per site in the best RAxML tree.

For further evaluation the interrelationships of Annelida sensu lato, we performed additional phylogenetic analyses with a smaller taxon set comprising 30 species (all species from the lophotrochozoan branch of the larger taxon set). ML analyses were done comparing mtREV (RaxML) and mtART (Treefinder) models; in addition a Bayesian analysis was performed with mtREV model (MrBayes). Myzostomida, Sipuncula and other Annelida formed a monophyletic group (Fig. 6) supported by ML bootstrapping (mtREV: 92%, mtART: 98%), but not by BI, where support is below 0.95 (Bayesian posterior probabilities). Sipuncula and Annelida together form a clade well supported by all three analyses, while Annelida without Sipuncula found best support only in BI, while the ML analyses do not significantly support this group, leaving open if there is a basal split between Sipuncula and the rest of the annelids. In the best ML-mtART tree *Platynereis *is found as sister to Sipuncula tree, but with bootstrap support below 50%. Well supported subtaxa of annelids are Pogonophora (s.lato), Clitellata, Pogonophora+Clitellata, Orbiniidae (*Scoloplos*+*Orbinia*). Topologies obtained in the three analyses differ in the position of *Urechis *(Echiura), which is found as sister to Maldanidae+Terebelliformia in the best ML tree with mtREV model (bootstrap support 65%), as sister to Orbiniidae in the best tree with mtART model (bootstrap support below 50%) and as sister to Pogonophora+Clitellata in BI (BPP below 0.95).

In addition we performed an AU test as implemented in CONSEL to statistically test the hypothesis of a sister group relationship between Sipuncula and Mollusca. We were able to significantly reject (p < 0.001) this hypothesis compared to the best ML-tree (mtREV).

## Conclusion

Annelida, in traditional phylogenetic systems the sister group to Arthropoda, are nowadays included in the taxon Lophotrochozoa by almost all large scale analyses [26,27,52-54]. In this view more and more molecular studies no longer support the monophyly of the classical Annelida ("polychaetes" and clitellates). As well as the unsegmented Pogonophora, Echiura, and Myzostomida the Sipuncula have also been under suspect to be included in what was called Annelida *sensu lato *[8,25,26,28]. The complete mitochondrial genomic sequence of *Sipunculus nudus *presented in this paper, adds an important piece of evidence to answer the question of sipunculid position in the metazoan tree of life. Our sequence data and gene order analysis clearly support an affinity of Sipuncula to Annelida *s. l*. (including Pogonophora, Echiura and Myzostomida) rather than to Mollusca or any other phylum. It still remains an open question if Sipuncula and the whole Annelida *s. l*. are sister groups (as the most parsimonious explanation of gene order data suggests), or if Myzostomids form the sister group to Sipuncula and the remaining Annelida (as sequence based analyses favour). In sequence-based analyses the myzostomid is the annelid taxon with the longest branch, suggesting a more rapid evolution of mitochondrial sequence in this taxon. Therefore analyses placing Myzostomids outside the Annelida are probably misleading due to higher substitution rates in myzostomids.

## Methods

### Animals, DNA purification

A specimen of *S. nudus *was collected in Concarneau, France and conserved in 100% ethanol. Using the DNeasy^® ^Blood & Tissue kit (Qiagen, Hilden, Germany) we followed the instructions given to extract DNA from animal tissues and used approximately 1 × 1 cm of the body wall from one individual.

### PCR and purification of DNA fragments

EST sequence fragments for the genes *nad1*, *nad3*, *rrnL*, *cob*, *cox1*, *cox2 *and *cox3 *were used to design the first species specific primer pairs [27]. The complete mitochondrial genome of *S. nudus *was amplified in PCR fragments generated with species specific primer pairs from EST information (see Table 2). All PCRs were done with Eppendorf Mastercycler or Eppendorf Mastercycler Gradient thermocyclers. PCRs were carried out in 50 μl volumes (41.75 μl water, 5 μl 10× buffer, 0.25 μl Taq polymerase (5 U/μl), 1 ml dNTP mixture, 1 μl template DNA, 1 μl primer mixture (10 μM each)) using the Eppendorf 5-prime kit (Eppendorf, Germany). The cycling conditions were as follows: 94°C for 2 min (initial denaturation); 40 cycles of 94°C for 30 sec (denaturation); primer-specific temperature (see Table 2) for 1 min (annealing), 68°C for 1 min (elongation), was followed by 68°C for 2 min (final elongation). After 40 cycles the samples were stored at 4°C and visualised on a 1% ethidium bromide-stained TBE agarose gel, respectively. DNA fragments expected to be larger than 3 kb, were amplified in 25 μl volumes (16.75 μl water, 2.5 μl buffer, 0.25 μl Takara LA Taq polymerase, 4 μl dNTP mixture, 1 μl template DNA, 0.5 μl primer mixture (10 μM each)) under the following long PCR conditions (Takara LA kit): 94°C for 2 min (initial denaturation); 40 cycles of 94°C for 30 sec (denaturation), primer-specific temperature for 1 min (annealing) and 72°C for 10 min (elongation). After the final elongation step (68°C for 2 min), samples were treated as described above. PCR products were purified with mini-spin columns provided in the Nucleo Spin Extract II kit (Macherey & Nagel) and the Blue Matrix PCR/DNA clean up DNA Purification kit (EurX, Gdansk, Poland). Dependent on the band intensity on the agarose gel, DNA was eluted in 30–60 μl elution buffer and stored at -20°C. Slightly contaminated samples were cut from a 1% ethidium bromide-stained TAE agarose gel and purified with the QIAquick Gel Extraction kit (Qiagen) afterwards.

**Table 2:** Primer pairs and corresponding annealing temperatures used for successful amplification of mitochondrial genome fragments from *Sipunculus nudus*

**Primer names**	**Primer sequence (5'-3')**	**Annealing temperature**	**Approxim. size of PCR product**
Sn-cox1-f	CTCCCACTTAGCACCCTC	48°C	400 bp
Sn-cox2-r	TAAGAGAATAATGGCGGG		
			
Sn-cox2-f	CCAACCACTCTTTTATGCC	50°C	700 bp
Sn-cox3-r	CCAGGATTAGGGCGGT		
			
Sn-cox3-f	TTTTCCTATACCTCTGCATC	48°C	700 bp
Sn-cob-r	TTGAATGACAAGGCAGAGA		
			
Sn-cob-f	CTCCTCGCCCCCAA	48°C	2500 bp
Sn-16S-r	GATTTATTGAAGAGTGGTTAGTGA		
			
Sn-16S-f	TCATACCCCGCACTCC	48°C	600 bp
Sn-nd3-r	CAAACCCGCACTCAAAC		
			
Sn-nd3-f	GGTAAGTGAACGGGGAAC	50°C	2500 bp
Sn-nd1-r	AAAAGGTTGGGGGAGG		
			
Sn-nd1-f	CGCTATCACCTCCACCTT	50°C	4000 bp
Sn-cox1-r	ATTCGGCACGGATAAGA		

### Cloning

If the DNA amount, obtained by PCR, turned out to be insufficient for sequencing, the respective fragment was cloned in a pGEM-T Easy Vector (Promega). Ligation was carried out in 5 μl volumes instead of the double amount, proposed in the protocol. In each case 2 μl of the sample were used for transformation in 50 μl competent *E. coli *XL Gold (Stratagene) cells. Colonies, containing recombinant plasmids, were detected via blue-white screen on LB selection plates, charged with IPTG, ampicillin and X-gal. To check whether the desired insert had been really transferred to the picked out colonies, a minimum amount of each colony (approximately half of it) was utilized as DNA template in a colony PCR. PCRs were run in 50 μl volumes (ingredients, amounts and conditions as above named), using M13F and M13R vector primers. Products were checked on 1% TBE agarose gels and – if they contained an insert of the anticipated size – transferred to LB/ampicillin medium. After proliferation over night, samples were purified according to the guidelines of the Quantum Prep-Kit (Bio Rad) and finally stored at -20°C.

### Sequencing and gene annotation

The amplified fragments were set up in 10 μl reaction volumes (2.5 μl DNA, 2.5 μl water, 1 μl primer (10 μM), 4 μl DCTS master mix) and sequencing PCR reactions were carried out according to the following procedure: 96°C for 20 sec (denaturation); primer-specific temperature for 20 sec (annealing); 60°C for 2 min (elongation). After 30 cycles the samples were sequenced with a CEQ™8000 capillary sequencer (Beckmann-Coulter) and the appropriate CEQ DCTS Quick Start kit (Beckmann-Coulter).

While the first checking of the sequences was carried out with the CEQ 8000 software (Beckman-Coulter), the actual sequence assemblage was done with BioEdit, version 7.0.5 [55]. Protein coding and ribosomal RNA genes, encoded in the mtDNA, were identified by BLAST (blastn, tblastx) searches on NCBI databases and by aligning the different sipunculid fragments with the mt genome of the echiurid *Urechis caupo*. To revise the final consensus sequence of *S. nudus*, further mt-genome data of relatively closely related taxa were retrieved from the OGRe database [56]. The species used for sequence comparison were: *Platynereis dumerilii *(Annelida), *Clymenella torquata *(Annelida), *Orbinia latreillii *(Annelida), *Lumbricus terrestris *(Annelida), *Terebratalia transversa *(Brachiopoda), *Terebratulina retusa *(Brachiopoda), *Laqueus rubellus *(Brachiopoda), *Urechis caupo *(echiura), *Epiperipatus biolleyi *(Onychophora), and *Flustrellidra hispida *(Bryozoa), see Table 3 for accession numbers. Transfer RNA genes and their putative secondary structures, were determined with the tRNAscan-SE [57] and ARWEN [58] and for the missing ones by eye inspection of candidate regions. The genome sequence was deposited in NCBI database [GenBank: FJ422961].

**Table 3:** Species, systematic position and accession number of mitochondrial genome sequences used in the phylogenetic analysis and/or for of gene order comparisons

**Species**	**Taxonomic position**	**Accession no**.
*Sipunculus nudus*	Sipuncula	FJ422961
Phascolopsis gouldii*	Sipuncula	AF374337
*Urechis caupo*	Echiura	NC_006379
Myzostoma seymourcollegiorum*	Myzostomida	EF506562
*Lumbricus terrestris*	Annelida – Clitellata	NC_001677
*Perionyx excavatus*	Annelida – Clitellata	NC_009631
Helobdella robusta*	Annelida – Clitellata	AF178678
*Platynereis dumerilii*	Annelida – "Polychaeta"	NC_000931
*Orbinia latreillii*	Annelida – "Polychaeta"	NC_007933
Eclysippe vanelli*	Annelida – "Polychaeta"	EU239687
*Clymenella torquata*	Annelida – "Polychaeta"	NC_006321
*Pista cristata*	Annelida – "Polychaeta"	NC_011011
*Terebellides stroemi*	Annelida – "Polychaeta"	NC_011014
Scoloplos armiger*	Annelida – "Polychaeta"	DQ517436
Galathealinum brachiosum*	Annelida – Pogonophora	AF178679
Riftia pachyptila*	Annelida – Pogonophora	AY741662
*Epiperipatus biolleyi*	Onychophora	NC_009082
*Limulus polyphemus*	Chelicerata – Xiphosura	NC_003057
*Heptathela hangzhouensis*	Chelicerata – Araneae	NC_005924
*Pseudocellus pearsei*	Chelicerata – Ricinulei	NC_009985
*Lithobius forficatus*	Myriapoda – Chilopoda	NC_002629
*Petrobius brevistylis*	Hexapoda – Archaeognatha	NC_007689
*Locusta migratoria*	Hexapoda – Orthoptera	NC_001712
*Artemia franciscana*	Crustacea – Anostraca	NC_001620
*Triops cancriformis*	Crustacea – Phyllopoda	NC_004465
*Penaeus monodon*	Crustacea – Decapoda	NC_002184
*Priapulus caudatus*	Priapulida	NC_008557
Cephalothrix rufifrons*	Nemertea	EF140788
Phoronis psammophila*	Phoronida	AY368231
*Terebratulina retusa*	Brachiopoda	NC_000941
*Laqueus rubellus*	Brachiopoda	NC_002322
*Terebratalia transversa*	Brachiopoda	NC_003086
*Katharina tunicata*	Mollusca – Polyplacophora	NC_001636
*Lottia digitalis*	Mollusca – Gastropoda	NC_007782
*Haliotis rubra*	Mollusca – Gastropoda	NC_005940
*Conus textile*	Mollusca – Gastropoda	NC_008797
*Ilyanassa obsoloeta*	Mollusca – Gastropoda	NC_007781
*Thais clavigera*	Mollusca – Gastropoda	NC_010090
*Lophiotoma cerithiformis*	Mollusca – Gastropoda	NC_008098
*Nautilus macromphalus*	Mollusca – Cephalopoda	NC_007980
*Octopus ocellatus*	Mollusca – Cephalopoda	NC_007896
*Venerupis phllippinarum*	Mollusca – Bivalvia	NC_003354
*Argopecten irradians*	Mollusca – Bivalvia	NC_009687
*Lampsilis ornata*	Mollusca – Bivalvia	NC_005335
*Siphonodentalium lobatum*	Mollusca – Scaphopoda	NC_005840
*Loxocorone allax*	Entoprocta	NC_010431
*Loxosomella aloxiata*	Entoprocta	NC_010432
*Flustrellidra hispida*	Bryozoa/Ectoprocta	NC_008192
*Paraspadella gotoi*	Chaetognatha	NC_006083
*Spadella cephaloptera*	Chaetognatha	NC_006386
*Brachionus plicatilis*	Rotifera	NC_010484
*Leptorhynchoides thecatus*	Acanthocephala	NC_006892
*Anisakis simplex*	Nematoda	NC_007934
*Agamermis sp*.	Nematoda	NC_008231
*Onchocercus volvulus*	Nematoda	NC_001861
*Caenorhabditis elegans*	Nematoda	NC_001328
*Trichinella spiralis*	Nematoda	NC_002681
*Xiphinema americanum*	Nematoda	NC_005928
Paratomella rubra*	Acoela	AY228758
Microstomum lineare*	Platyhelminthes – "Turbellaria"	AY228756
*Fasciola hepatica*	Platyhelminthes – "Trematoda"	NC_002546
*Paragoniums westermanni*	Platyhelminthes – "Trematoda"	NC_002354
*Gyrodactylus salaris*	Platyhelminthes – "Trematoda"	NC_008815
*Microcotyle sebastis*	Platyhelminthes – "Trematoda"	NC_009055
*Schistosoma haematobium*	Platyhelminthes – "Trematoda"	NC_008074
*Trichobilharzia regenti*	Platyhelminthes -	NC_009680
*Hymenolepis diminuta*	Platyhelminthes – Cestoda	NC_002767
*Taenia asiatica*	Platyhelminthes – Cestoda	NC_004826
*Echinococcus granulosus*	Platyhelminthes – Cestoda	NC_008075
*Balanoglossus carnosus*	Enteropneusta	NC_001887
*Saccoglossus kowalevskii*	Enteropneusta	NC_007438
*Florometra serratissima*	Echinodermata – Crinoidea	NC_001878
*Paracentrotus lividus*	Echinodermata – Echinoidea	NC_001572
*Xenoturbella bocki*	Xenoturbellida	NC_008556
*Acropora tenuis*	Cnidaria – Anthozoa	NC_003522
*Metridium senile*	Cnidaria – Anthozoa	NC_000933

*: incomplete genome sequence

### Phylogenetic analysis

The amino acid alignments of the protein-coding genes (except the two short and highly variable genes *atp8 *and *nad4L*) were concatenated. Sequence data from 74 species were included in the large analyses (see Table 3 for all species names and accession numbers). The tree was rooted with two representatives of Cnidaria. Maximum likelihood analysis was performed with RAxML, ver. 7.00 [59,60]. mtREV+G+I was chosen as model for aminoacid substitutions. The complete dataset was partitioned, so that model parameters and amino acid frequencies were optimized for each single gene alignment. 100 bootstrap replicates were performed to infer the support of clades from the best tree. A second set of analyses were done with a reduced dataset of 30 species. This dataset was analyzed with RAxML as described above (model mtREV+G+I, partitioned according to the 12 single gene sequences), with 1000 bootstrap replicates. Secondly we did a Bayesian analysis with MrBayes ver. 3.1.2 [61]. In BI the mtREV+G+I model was used and 1.000.000 generations were run with 8 chains in parallel. Trees were sampled every 1000 generations, while the first 200 trees were discarded as burn-in (according to the likelihood plot). In addition we performed a ML analysis using the mtART+G+I model with Treefinder [62] and "edge support" analysis, again with a partitioned dataset (= independently optimizing model parameters for the 12 genes).

For comparison of the hypothesis that sipunculids might be closely related with molluscs and our best tree, we used a constraint for a ML-analysis (Sipuncula + Mollusca) of the sequence dataset using RaxML [59] with parameters described above. We computed per-site log-likelihoods with RAxML for both topologies (best tree and constrained topology) and conducted an au-test as implemented in CONSEL [63].

## Abbreviations

*atp6 *and *8*: genes encoding ATPase subunit 6 and 8; bp: base pairs; bt: bootstrap; *cox 1–3*: genes encoding cytochrome oxidase subunits I-III; *cob*: gene encoding cytochrome b; BI: Bayesian Inference; ML: Maximum Likelihood; mtDNA: mitochondrial DNA; mt-genome: mitochondrial genome; *nad1-6 *and *nad4L*: genes encoding NADH dehydroenase subunits 1–6 and 4L; PCR: polymerase chain reaction; rRNA: ribosomal RNA; *rrnL*: large rRNA subunit (16S); *rrnS*: small rRNA subunit (12S); tRNA: transfer RNA; *trnX*: tRNA gene (*X *is replaced by one letter amino acid code).

## Authors' contributions

LP and TB conceived and supervised this study. AcM and BL constructed the EST libarary. AdM did all PCR experiments and sequencing of the mitochondrial genome. AdM and LP annotated the genome. AdM, LP and CB performed phylogenetic analysis of sequence data, LP analyzed gene order data. AdM and LP wrote the main part of the manuscript, all other authors helped in interpretation of data and discussion of results.

## Supplementary Material

Additional File 1 **Full version of figure**2. Secondary structure of tRNAs identified in the mitochondrial genome of *S. nudus*. The best found putative secondary structure of tRNA-Cys (box) seems to be strongly derived, probably non-functional or subject to gene editing. Click here for file
