# Supplementary figures and images for: Mitochondrial genome sequence and gene order of Sipunculus nudus give additional support for an inclusion of Sipuncula into Annelida
Source: BMC Genomics. 2009 Jan 16;10:27. doi: 10.1186/1471-2164-10-27 (PMC2639372; doi:10.1186/1471-2164-10-27)

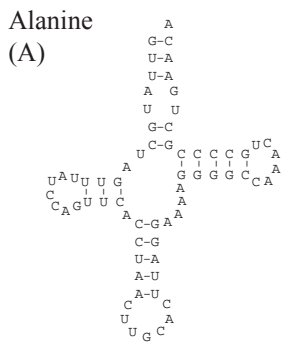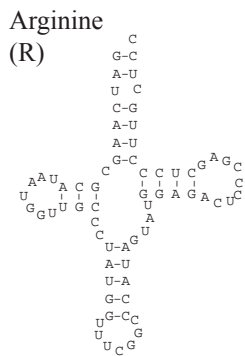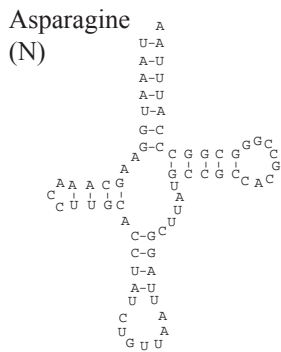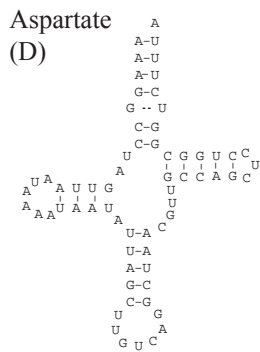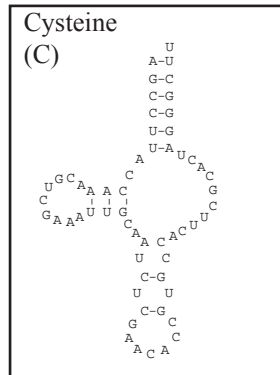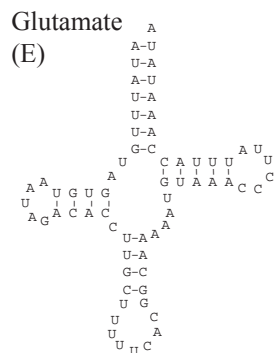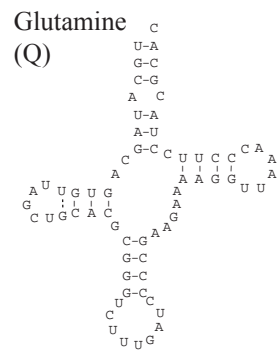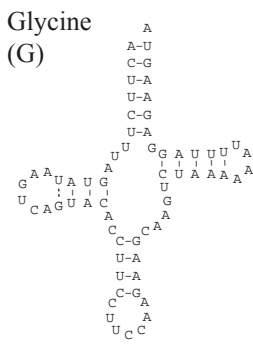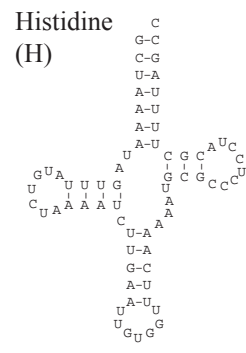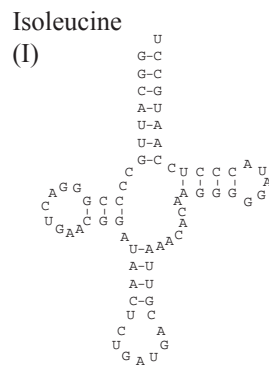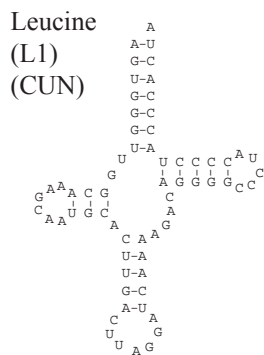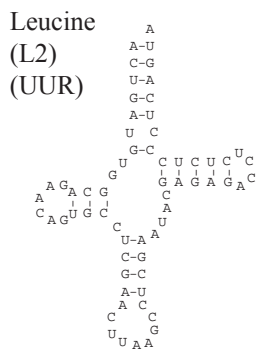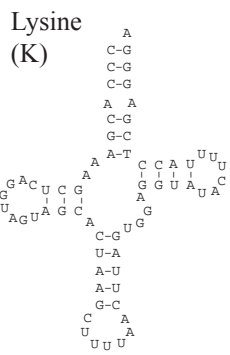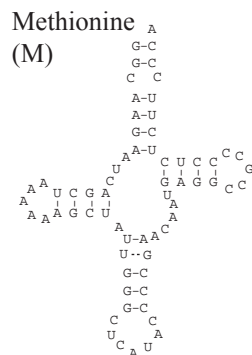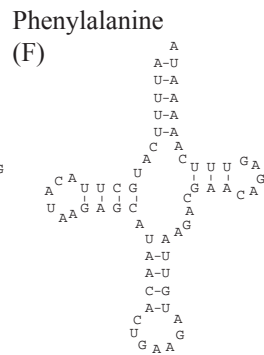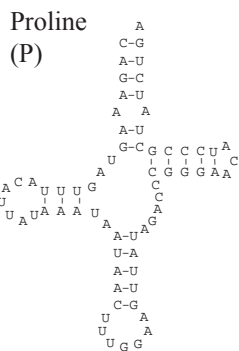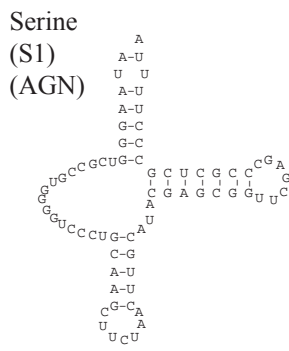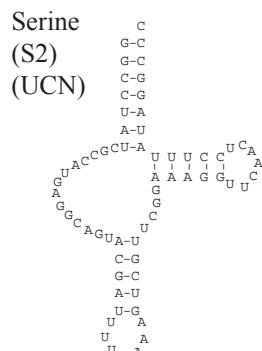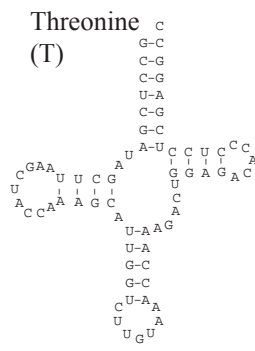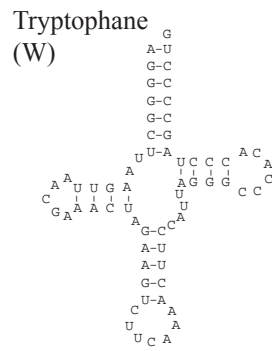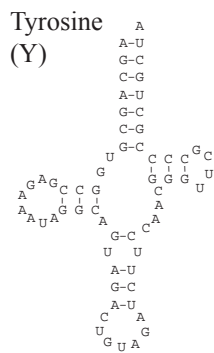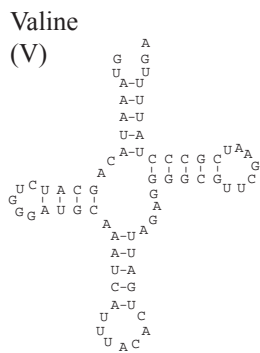

Supplement: Additional File 1 — Full version of figure2. Secondary structure of tRNAs identified in the mitochondrial genome of S. nudus. The best found putative secondary structure of tRNA-Cys (box) seems to be strongly derived, probably non-functional or subject to gene editing. [file 1471-2164-10-27-S1.pdf]
